# The Effect of Temperature Variations on the 5-Hydroxymethylfurfural Content, Antioxidant and Antimicrobial Properties of Honey

**DOI:** 10.3390/life16081366

**Published:** 2026-08-19

**Authors:** Guntima Suwannapong, Mark Eric Benbow, Rujira Ponkit, Worachote Boonsriwong, Sanchai Naree

**Affiliations:** 1 Department of Biology, Faculty of Science, Burapha University, Chon Buri 20131, Thailand; beefambuu@gmail.com (G.S.); br_27877@hotmail.com (R.P.); 2 Department of Entomology, Michigan State University, East Lansing, MI 48824, USA; eric.benbow@gmail.com; 3 Department of Osteopathic Medical Specialties, Michigan State University, East Lansing, MI 48824, USA; 4 Pharmacology and Biopharmaceutical Department, Faculty of Pharmaceutical Sciences, Burapha University, Chon Buri 20131, Thailand; worachote@go.buu.ac.th

**Keywords:** antibacterial activity, antioxidant activity, *Apis dorsata*, honey, 5-hydroxymethylfurfural, temperature regimes

## Abstract

*Apis dorsata* honey is a rich source of natural antioxidants and antibacterial compounds. Thermal processing is commonly used to improve honey fluidity and delay crystallization; however, it may also affect bioactive properties and promote the formation of 5-hydroxymethylfurfural (5-HMF). We evaluated the impact of temperature regimes on biological activities and 5-HMF contents of *A. dorsata* honey. Honey samples were collected from *A. dorsata* colonies. Honey color, total phenolic content (TPC), total flavonoid content (TFC), 5-HMF content, and antibacterial activity against *Escherichia coli* and *Staphylococcus aureus* were investigated after being treated at room temperature, 55 °C, 65 °C, 75 °C, and 85 °C for 0.5, 1, and 2 h. Heating at 55 °C, 65 °C, 75 °C, and 85 °C significantly enhanced color intensity, TPC, and TFC. Samples treated at 85 °C for two hours showed the highest antioxidant activity, with an EC_50_ significantly lower than that of the others (51.31 ± 0.77 mg/mL). Conversely, 5-HMF content increased markedly with increasing exposure temperature and time. Antibacterial assays revealed that all treated samples inhibited *E. coli*, whereas no inhibitory activity was detected against *S. aureus*. These results define important processing parameters in food safety for heating honey to preserve bioactivity integrity without loss in quality and maintenance of product stability and sustainability.

## 1. Introduction

Honey is a high-value organic product containing nutritional and medicinal properties produced by foraging honey bees [1,2]. In addition to being a sweetener, honey exhibits significant biological activities that can be attributed to extensive phytochemical components, especially phenolic acids, flavonoids, and other antioxidant-related compounds [3,4]. These bioactive components are responsible not only for the health-related effects of honey but can also be used as quality indicators to distinguish different types and botanical origins of honeys [5,6]. Honey produced by different *Apis* species exhibits significant variation in color, aroma, taste, and physicochemical properties, reflecting differences in entomological origin, foraging behavior, and environmental conditions [7,8,9,10].

The giant honey bee (*Apis dorsata*) is a wild species widely distributed throughout South and Southeast Asia, including Thailand, where its honey is highly valued by local communities as a traditional health-promoting food [2]. Unlike *A. mellifera*, which is managed in artificial hives, *A. dorsata* forages in natural forest ecosystems with access to diverse floral resources, producing honey with distinct physicochemical and bioactive characteristics [2,11,12,13]. These characteristics make *A. dorsata* honey a functional food and, furthermore, a potential natural antioxidant and antibacterial composition applicable in sustainable health areas [14]. However, processing and storage substantially affect the quality and biological activities of honey [15,16]. *Apis dorsata* has a larger foraging area than *A. mellifera,* which can produce honey from diverse floral sources, resulting in distinct physicochemical characteristics and bioactive compositions compared with the commercially managed *A. mellifera* [2]. Several studies have reported that *A. dorsata* honey possesses higher total phenolic content, total flavonoid content, stronger antioxidant capacity, and greater antimicrobial activity than many commercially produced *A. mellifera* honeys, although these characteristics vary according to geographical origin and floral source [17,18].

Heating is a routine operation in honey handling and processing, which serves to delay crystallization and increase its fluidity for packing and consumer application [19,20]. Despite the technological advantages, thermal treatment affects the quality of honey by causing loss of heat-sensitive phytochemicals, reducing antibacterial activity [21,22,23]. However, there are many studies that have shown that heating honey affects enzymatic properties, antimicrobial activities and the contents of phenolic and flavonoid compounds [20,24,25]. Moreover, the high temperatures induce the formation of 5-HMF or 5-hydroxymethylfurfural [20,26,27], which is a cyclic aldehyde formed by fructose and glucose dehydration in an acidic environment, such as honey [28,29]. This major honey quality factor is an indicator of honey freshness and overheating. In fresh honeys, there is practically low 5-HMF, but it increases upon storage, depending on storage temperature [23,30]. The 5-HMF content after processing shall not be more than 40 mg/kg, but in the case of honey of declared origin from countries with tropical ambient temperatures, including Thailand, the 5-HMF content shall not be more than 80 mg/kg [31].

Elevated levels of 5-HMF are undesirable due to their reported cytotoxic, mutagenic, and potential carcinogenic effects, thereby raising significant concerns regarding food safety and public health [32,33,34]. Although there have been many studies concerning the effect of heating processes on honey quality, which mainly investigate important types of honey, especially those made by *A. mellifera*, relatively little work has been done on *A. dorsata* honey. However, the response of *A. dorsata* honey to heat treatment is still not adequately characterized, particularly with respect to how these treatments should be conducted to retain desired antioxidant and antimicrobial activity while reducing 5-HMF production. This knowledge is important for the safe processing of *A. dorsata* honey while maintaining biological quality. Thus, in this research, the effects of heat treatment of *A. dorsata* honey at room temperature, 55 °C, 65 °C, 75 °C, and 85 °C for timings of 0.5, 1, and 2 h were comprehensively investigated. Color analysis was analyzed using the spectrophotometric method, total phenolic contents (TPC) were evaluated by the Folin–Ciocalteu method, total flavonoid contents (TFC) were measured by the aluminum chloride colorimetric method, antioxidant activity was determined using the 2,2-diphenyl-1-picrylhydrazyl (DPPH) radical scavenging assay, 5-HMF contents were estimated using the spectrophotometric method, and antibacterial activity against *Escherichia coli* ATCC 25922 and *Staphylococcus aureus* ATCC 6538 was investigated using the agar well diffusion method.

This study provides practical guidance in food safety for optimizing thermal processing of *A. dorsata* honey, allowing producers to enhance antioxidant properties while maintaining product quality. It also offers valuable scientific insight into balancing bioactivity preservation with 5-HMF control, supporting safer consumption and improved honey processing standards.

## 2. Materials and Methods

### 2.1. Honey Samples

Honey samples were collected from three healthy colonies of *A. dorsata* located in Samut Songkhram province, Thailand, in December 2024 [13°25′09.3″ N 99°57′16.7″ E]. The samples were stored in airtight containers at 4 °C until further analysis. The experiment was performed at Bee Health and Their Hive Products for Supporting SDGs Research Unit, Department of Biology, Faculty of Science, Burapha University, Thailand, and Faculty of Pharmaceutical Sciences, Burapha University, Thailand.

### 2.2. Heat Exposure and Sample Preparation

The honey was divided into five groups (50 g/group) from each colony into a 100 mL glass bottle with a screw cap (Duran, DWK Life Sciences GmbH, Wertheim, Germany), and subjected to thermal treatment at 55 °C, 65 °C, 75 °C, and 85 °C for 0.5, 1, and 2 h in a hot-air oven (Binder, ED53, BINDER GmbH, Tuttlingen, Germany). A control group was maintained at room temperature without heat treatment. After heating, samples were cooled to room temperature, then diluted with distilled water to final concentrations of 20%, 10%, 5%, 2.5%, and 1.25% *w*/*v* of honey solutions and stored at 4 °C until further analysis.

### 2.3. Color Analysis

The color of the honey samples was determined using microplate readers (Therma Scientific Multiskan GO, Thermo Fisher Scientific, Tokyo, Japan). One milliliter of 50% *w*/*v* honey solution was centrifuged at 6000 *g* for 5 min. The absorbance of supernatants was measured at 635 nm and converted into mm Pfund using the following equation [35]. The color (mm Pfund) was interpreted using the criteria following the U.S. Department of Agriculture classifications (˂9 = water white; 9 to 17 = extra white; 18 to 34 = white; 35 to 50 = extra light amber; 51 to 85 = light amber; 86 to 144 = amber; ˃114 = dark amber) [36].

Pfund (mm) = −38.7 + 371.39 × Abs

where Pfund = the honey color value on the Pfund scale (mm); Abs = the value of the absorbance read at the wavelength 635 nm.

### 2.4. Total Phenolic Content (TPC) Analysis

TPC was determined using the Folin–Ciocalteu method [12]. Twenty microliters of 20% *w*/*v* honey solution were mixed with 100 µL of 0.2 N Folin–Ciocalteu reagent (Sigma-Aldrich, Sigma-Aldrich, Inc., St. Louis, MO, USA) in a 96-well cell plate. The plate was kept in the dark at room temperature (25 °C) for 5 mins. Then, 80 µL of 0.75% *w*/*v* sodium carbonate (Na_2_CO_3_) (Kemaus, Elago Enterprises Pty Ltd., Sydney, New South Wales, Australia) was added and heated in an incubator (Memmert, UN 55, Memmert GmbH, Schwabach, Germany) at 40 °C for 20 mins. The plates were kept on ice at 4 °C for 5 min. Absorbance was measured at 760 nm using a microplate spectrophotometer (Thermo Scientific, Multiskan GO, Thermo Fisher Scientific, Tokyo, Japan). Distilled water was used as a blank. Five concentrations of gallic acid (Sigma-Aldrich, Sigma-Aldrich, Inc., MO, USA) (31.25, 62.5, 125, 250, and 500 µg/mL) were used as the standard, and results were expressed as mg gallic acid equivalents (GAE) per 100 g of honey.

### 2.5. Total Flavonoid Content (TFC) Analysis

TFC was estimated using the aluminum chloride colorimetric method [12]. Twenty microliters of 20% *w*/*v* honey solution were mixed with 80 µL of distilled water and 6 µL of 5% *w*/*v* sodium nitrite (NaNO_2_) (Kemaus, Elago Enterprises Pty Ltd., New South Wales, Australia) in a 96-well cell plate. The plate was kept in the dark at room temperature (25 °C) for 5 mins. Then, 6 µL of 10% *w*/*v* aluminum chloride (AlCl_3_) (Kemaus, Elago Enterprises Pty Ltd., New South Wales, Australia) was added to a 96-well cell plate. The plate was kept in the dark at room temperature (25 °C) for 5 mins. Forty microliters of 1 M sodium hydroxide (NaOH) (Merck, Merck KGaA, Darmstadt, Germany) and 48 µL of distilled water were added to a 96-well cell plate. The plate was kept in the dark at room temperature (25 °C) for 30 mins. Absorbance was measured at 430 nm using a microplate spectrophotometer (Thermo Scientific, Multiskan GO, Thermo Fisher Scientific, Tokyo, Japan). Distilled water was used as a blank. Five concentrations of quercetin (Sigma-Aldrich, Sigma-Aldrich, Inc., MO, USA) (31.25, 62.5, 125, 250, and 500 µg/mL) were used as standards, and results were expressed as mg quercetin equivalents (QE) per 100 g of honey.

### 2.6. 2,2-Diphenyl-1-Picrylhydrazyl (DPPH) Radical Scavenging Assay

Antioxidant activity was determined using the 2,2-diphenyl-1-picrylhydrazyl (DPPH) radical scavenging assay, following the method described by Brand-Williams et al. (1995) [37]. Fifty microliters of each sample (20%, 10%, 5%, 2.5%, and 1.25% *w*/*v* of honey solutions) were mixed with 150 µL of 100 µM DPPH solution (Sigma-Aldrich, Sigma-Aldrich, Inc., MO, USA) in a 96-well cell plate. The plate was kept in the dark at room temperature (25 °C) for 30 mins. Absorbance was measured at 517 nm using a microplate spectrophotometer (Thermo Scientific, Multiskan GO, Thermo Fisher Scientific, Tokyo, Japan). Absolute ethanol was used as a blank. The antioxidant capacity was expressed as the percentage of DPPH inhibition (%DPPH inhibition), which was determined using the following equation. Half maximal effective concentration (EC_50_) was calculated from the graph plotting the percentage of radical scavenging activity against honey sample concentration.

%DPPH inhibition = (A blank − A sample)/A blank

where A blank = absorbance of the absolute ethanol; A sample = absorbance of the sample.

### 2.7. 5-Hydroxymethylfurfural (5-HMF) Determination

All treatments were examined for 5-HMF content using spectrophotometric methods [12,38]. Honey samples (5 g) were dissolved in distilled water (25 mL) and transferred into a 50 mL volumetric flask. Subsequently, the honey solution was added 0.5 mL of Carrez I reagent (15 g potassium hexacyanoferrate (II) trihydrate (Loba Chemie, Loba Chemie Pvt. Ltd., Mumbai, Maharashtra, India) dissolved in distilled water and made up to 100 mL with distilled water) and 0.5 mL of Carrez II reagent (30 g zinc acetate dihydrate (Loba Chemie, Loba Chemie Pvt. Ltd., Maharashtra, India) dissolved in distilled water and made up to 100 mL with distilled water), then adjusted its volume to 50 mL with distilled water. Then, the solution was homogenized and filtered through filter paper Whatman paper no. (Whatman, GE Healthcare Life Sciences, Shanghai, China). The filtrate (5 mL) was then decanted into two flasks. One of the flasks contained 5 mL of distilled water (sample solution), whereas the other contained 5 mL of 0.2% sodium bisulfate (Loba Chemie, Loba Chemie Pvt. Ltd., Maharashtra, India) (reference solution). The absorbance of those solutions was measured by microplate spectrophotometer at wavelengths of 284 and 336 nm (Thermo Scientific Multiskan GO, Thermo Fisher Scientific, Tokyo, Japan). The 5-HMF content in honey was calculated using the following equation.

5-HMF = (A_284_ − A_336_) × 149.7 × 5 × D/W [mg/kg]

where A_284_ and A_336_ = the absorbances at 284 and 336 nm, respectively; the constant 149.7 = (126 × 1000 × 1000)/(16,830 × 10 × 5) is constant, 126 is molecular weight of 5-HMF, 16,830 is molar absorptivity ε of 5-HMF at λ = 284 nm, 1000 is conversion g into mg, 10 is conversion 5 into 50 mL, 1000 is conversion g of honey into kg, 5 is the theoretical nominal sample weight; D is the dilution factor (if applied); and W is the honey sample weight (g).

### 2.8. Agar Well Diffusion Assay

The antibacterial activity was investigated using the agar well diffusion method [39]. Gram-negative bacteria, *Escherichia coli* ATCC 25922, and Gram-positive bacteria, *Staphylococcus aureus* ATCC 6538, were grown on tryptic soy agar (TSA) (Himedia, HiMedia Laboratories, Mumbai, Maharashtra, India) at 37 °C for 24 h. The bacteria were resuspended in 5 mL of 0.85% *w*/*v* sodium chloride (NaCl) until the turbidity matched a 0.5 McFarland standard (1.5 × 10^8^ CFU/mL). The bacterial suspension was swabbed onto a Mueller–Hinton agar (MHA) (Himedia, HiMedia Laboratories, Maharashtra, India) plate using a sterilized cotton swab. After spreading, the plates were allowed to dry for at least 15 min. Then, a hole with a diameter of 6 mm was punched aseptically with a sterile cork borer, and 50 µL of the honey samples were transferred into the well. Then, agar plates were kept in an incubator (Memmert, UN 55, Memmert GmbH, Schwabach, Germany) at 37 °C for 24 h. Tetracycline (1 μg/μL) was used as a positive control. Each sample was tested in triplicate, and antibacterial activity was evaluated by measuring and recording the inhibition zone in mm.

### 2.9. Broth Microdilution Assay

A hundred micrograms of honey samples were two-fold serially diluted in a 96-well cell plate containing Mueller–Hinton broth (MHB) (Himedia, HiMedia Laboratories, Maharashtra, India) to get final concentrations of 500, 250, 125, 62.5, 31.25, and 15.625 μg/mL of honey solution. Fifty microliters of each bacterial suspension (1.5 × 10^6^ CFU/mL) were added into each well. The plates were kept in an incubator (Memmert, UN 55, Memmert GmbH, Schwabach, Germany) at 37 °C for 24 h. Then, the bacterial growth was observed. The minimum inhibitory concentration (MIC) was the lowest concentration of honey that resulted in no visible growth (clearly) in the well of a 96-well cell plate. The well containing no visible growth after MIC tests was inoculated on TSA (Himedia, HiMedia Laboratories, Maharashtra, India). Then, the plates were kept in an incubator (Memmert, UN 55, Memmert GmbH, Schwabach, Germany) at 37 °C for 24 h. The absence of growth in the recovery medium was evidence of the minimum bactericidal concentration (MBC).

### 2.10. Statistical Analysis

The data on color (mm Pfund) and total flavonoid contents of all treatments were not normally distributed (Jarque–Bera JB test: *p* < 0.05) but had equal variances (Levene’s test: *p* > 0.05). Non-parametric data were analyzed using a Kruskal–Wallis test followed by Mann–Whitney pairwise tests. In addition, the data for total phenolic content and 5-HMF contents of all treatments were normally distributed (Jarque–Bera JB test: *p* > 0.05) and had equal variances (Levene’s test: *p* > 0.05); thus, these variables were analyzed using a one-way ANOVA followed by Mann–Whitney pairwise tests. Moreover, the data for half maximal effective concentration (EC_50_) were normally distributed (Jarque–Bera JB test: *p* > 0.05) but had unequal variances (Levene’s test: *p* < 0.05), and so analyses used Kruskal–Wallis tests followed by Mann–Whitney pairwise tests.

## 3. Results

### 3.1. Color Analysis

**Figure 1:**
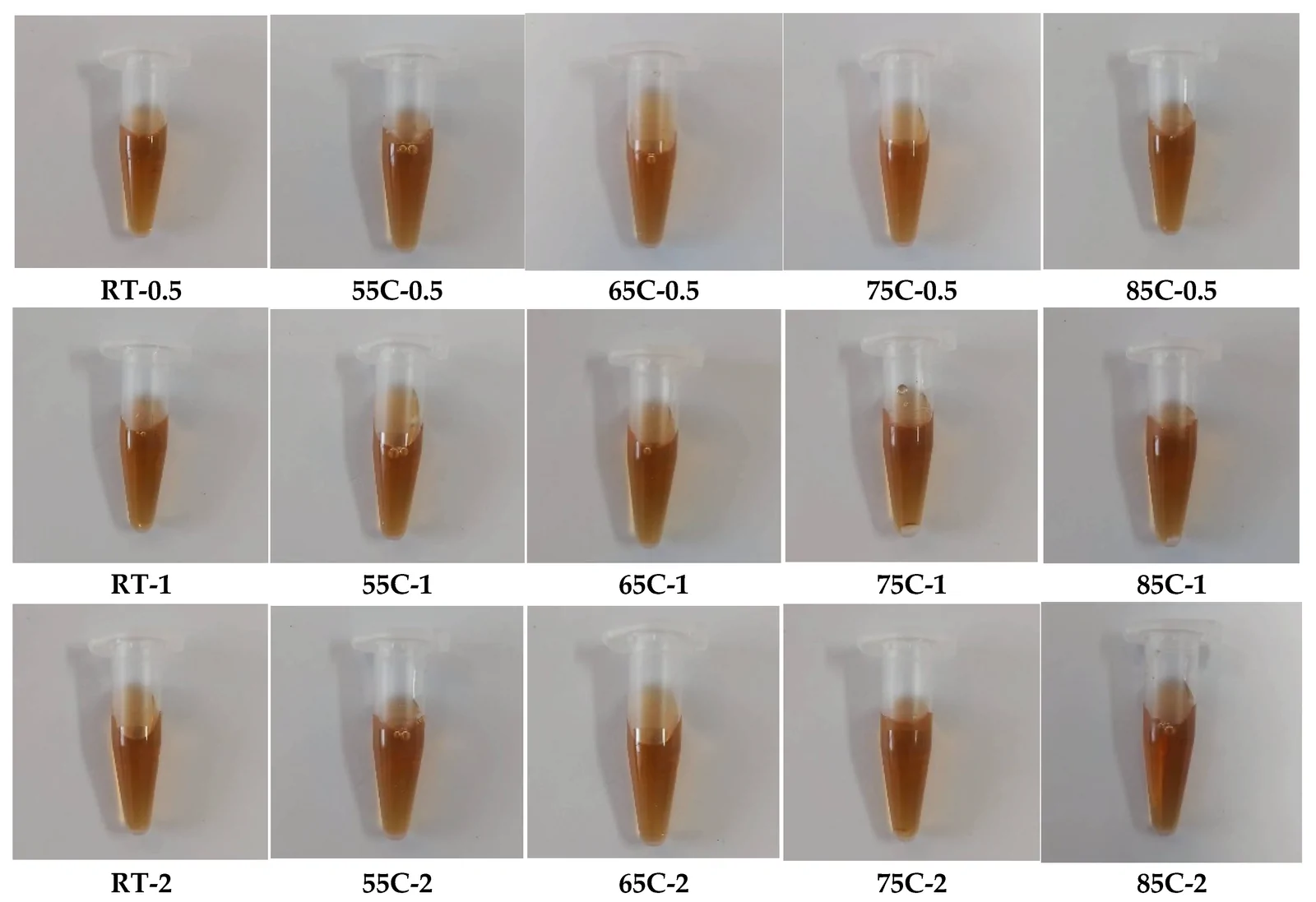
*Apis dorsata* honey from the fifteen treatment groups. The color (mm Pfund) was interpreted using the criteria following the U.S. Department of Agriculture classifications (˂9 = water white; 9 to 17 = extra white; 18 to 34 = white; 35 to 50 = extra light amber; 51 to 85 = light amber; 86 to 144 = amber; ˃114 = dark amber) [30]. The color of all treatments was light amber. RT-0.5, RT-1, and RT-2 (room temperature for 0.5, 1, and 2 h); 55C-0.5, 55C-1, and 55C-2 (55 °C for 0.5, 1, and 2 h); 65C-0.5, 65C-1, and 65C-2 (65 °C for 0.5, 1, and 2 h); 75C-0.5, 75C-1, and 75C-2 (75 °C for 0.5, 1, and 2 h); 85C-0.5, 85C-1, and 85C-2 (85 °C for 0.5, 1, and 2 h).

The color (mm Pfund) of *A. dorsata* honey heat at 65 °C, 75 °C, and 85 °C was significantly higher than that of 55 °C and room temperature at all three duration times (0.5, 1, and 2 h) (*χ*^2^ = 67.29, *df* = 14, *p* < 0.0001; Table 1); however, all their colors were identified as light amber (Figure 1) using the criteria following the U.S. Department of Agriculture classifications. The highest color (mm Pfund) of *A. dorsata* honey at a 0.5 h duration time of heat exposure was found in honey heated at 85 °C (85C-0.5) with 78.73 ± 0.43 mm Pfund, followed by honey from 75C-0.5, 65C-0.5, 55C-0.5, and RT-0.5, respectively. The same general trend continued at 1 and 2 h of heat exposure.

### 3.2. Total Phenolic Content (TPC) Analysis

TPC increased over time after heat exposure. The TPC of *A. dorsata* honey heated at 85 °C, 75 °C, and 65 °C was significantly higher than that of 55 °C and room temperature at all three duration times (0.5, 1, and 2 h) (*F* = 449.8, *df* = 14, *p* < 0.0001; Table 1). The highest TPC of *A. dorsata* honey at a 0.5 h duration time of heat exposure was found in honey heated at 85 °C (85C-0.5) with 875.86 ± 2.83 mg GAE/100 g, followed by honey from 75C-0.5, 65C-0.5, 55C-0.5, and RT-0.5, respectively. The same general trend continued at 1 and 2 h of heat exposure. See Table 1 for statistical comparisons between the groups within a duration time of heat exposure.

### 3.3. Total Flavonoid Content (TFC) Analysis

TFC increased over time after heat exposure. The TFC of *A. dorsata* honey heated at 85 °C, 75 °C, and 65 °C was significantly higher than that of 55 °C and room temperature at all three duration times (0.5, 1, and 2 h) (*χ*^2^ = 56.61, *df* = 14, *p* < 0.0001; Table 1). The highest TFC of *A. dorsata* honey (mean ± SE) at a 0.5 h duration time of heat exposure was found in honey heated at 75 °C (75C-0.5) with 656.12 ± 14.46 mg QE/100 g, followed by honey from 85C-0.5, 65C-0.5, RT-0.5, and 55C-0.5, respectively. The same general trend continued at 1 and 2 h of heat exposure.

### 3.4. DPPH Radical Scavenging Assay: Half Maximal Effective Concentration (EC_50_)

The half maximal effective concentration (EC_50_) decreased over time after heat exposure. The EC_50_ of *A. dorsata* honey heated at 85 °C, 75 °C, and 65 °C was significantly lower than that of room temperature at all three duration times (0.5, 1, and 2 h) (*χ*^2^ = 64.69, *df* = 14, *p* < 0.0001; Table 1). The highest antioxidant activity of *A. dorsata* honey (mean ± SE) at a 0.5 h duration time of heat exposure was found in honey heated at 85 °C (85C-0.5) with 62.67 ± 0.37 mg/mL, followed by honey from 75C-0.5, 65C-0.5, 55C-0.5, and RT-0.5, respectively. The same general trend continued at 1 and 2 h of heat exposure. See Table 1 for statistical comparisons between the groups within a duration time of heat exposure. Additionally, % DPPH inhibition increased over time after heat exposure. See Table S1 in Supplementary Data.

### 3.5. 5-Hydroxymethylfurfural (5-HMF) Determination

5-HMF content increased over time after heat exposure. The 5-HMF contents of *A. dorsata* honey heated at 85 °C, 75 °C, and 65 °C were significantly higher than those of 55 °C and room temperature at all three duration times (0.5, 1, and 2 h) (*F* = 54.89, *df* = 14, *p* < 0.0001; Table 1). The highest 5-HMF content of *A. dorsata* honey (mean ± SE) at a 0.5 h duration time of heat exposure was found in honey heated at 85 °C (85C-0.5) with 51.6 ± 1.76 mg/kg followed by honey from 75C-0.5, 65C-0.5, 55C-0.5, and RT-0.5, respectively. The same general trend continued at 1 and 2 h of heat exposure. See Table 1 for statistical comparisons between the groups within a duration time of heat exposure.

### 3.6. Pearson’s Correlation Analysis

**Figure 2:**
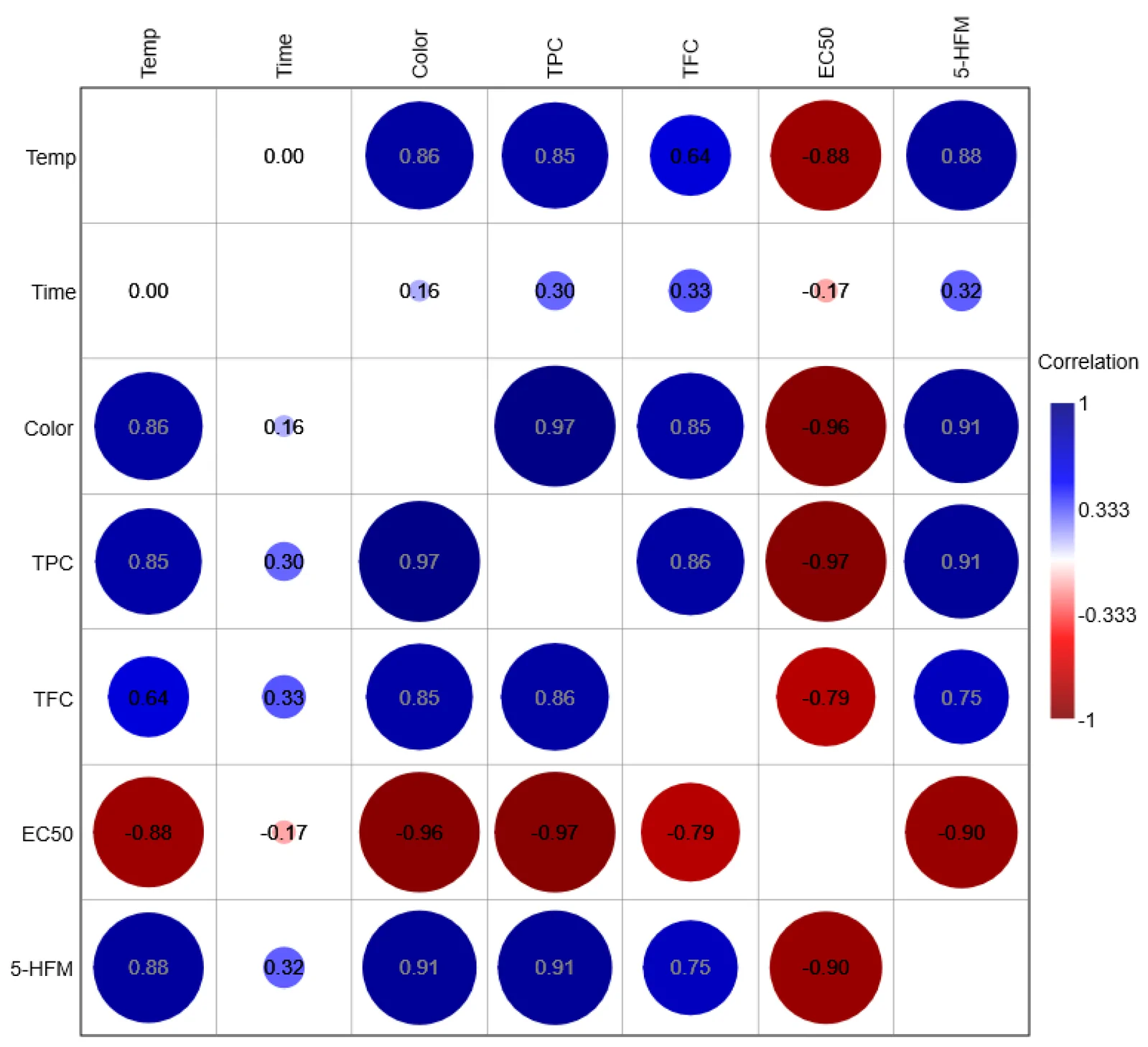
Pearson’s correlation analysis revealed strong interrelationships among temperature (Temp), heating time (Time), color, total phenolic content (TPC), total flavonoid content (TFC), antioxidant activity (EC_50_), and 5-hydroxymethylfurfural (5-HMF) contents of *A. dorsata* honey from the fifteen treatment groups. The correlation coefficient (r) ranges from –1 (a perfect negative correlation) to 1 (a perfect positive correlation); 0 = no correlation, 0–0.2 = very weak correlation, 0.2–0.4 = weak correlation, 0.4–0.6 = moderate correlation, 0.6–0.8 = strong correlation, 0.8–1.0 = very strong correlation.

Temperature was strongly and positively correlated with color (r = 0.86), TPC (r = 0.85), and 5-HMF (r = 0.88), indicating that increasing temperature markedly enhanced browning, phenolic content, and 5-HMF formation (Figure 2).

Temperature was also positively correlated with TFC (r = 0.64). Conversely, temperature was strongly negatively correlated with EC_50_ (r = −0.88), suggesting that higher temperatures were associated with lower EC_50_ values and consequently higher antioxidant activity (Figure 2). Heating time in this study (0.5, 1, and 2 h) was weakly correlated with color (r = 0.16), TPC (r = 0.30), TFC (r = 0.33), and 5-HMF (r = 0.32). These findings indicate that heating time for 0.5, 1, and 2 h had a smaller influence on the measured parameters than temperature. Color had strong positive correlations with TPC (r = 0.97), TFC (r = 0.85), and 5-HMF (r = 0.91). In contrast, color was strongly negatively correlated with EC_50_ (r = −0.96), indicating that darker honey samples generally possessed greater antioxidant activity. TPC exhibited very strong positive correlations with TFC (r = 0.86) and 5-HMF (r = 0.91), whereas it showed a very strong negative correlation with EC_50_ (r = −0.97), representing the strongest negative relationship observed in the analysis. This result suggests that higher phenolic content was closely associated with improved antioxidant activity. Similarly, TFC was positively correlated with 5-HMF (r = 0.75) and negatively correlated with EC_50_ (r = −0.79), indicating that flavonoid accumulation was positively associated with 5-HMF formation and antioxidant capacity. Finally, the strong negative correlation between EC_50_ and 5-HMF (r = −0.90) reflects the temperature-dependent nature of both processes: heating enhances antioxidant activity while simultaneously accelerating 5-HMF formation. However, this does not imply that 5-HMF itself is responsible for the increased antioxidant capacity.

### 3.7. Antibacterial Activity Assay

**Figure 3:**
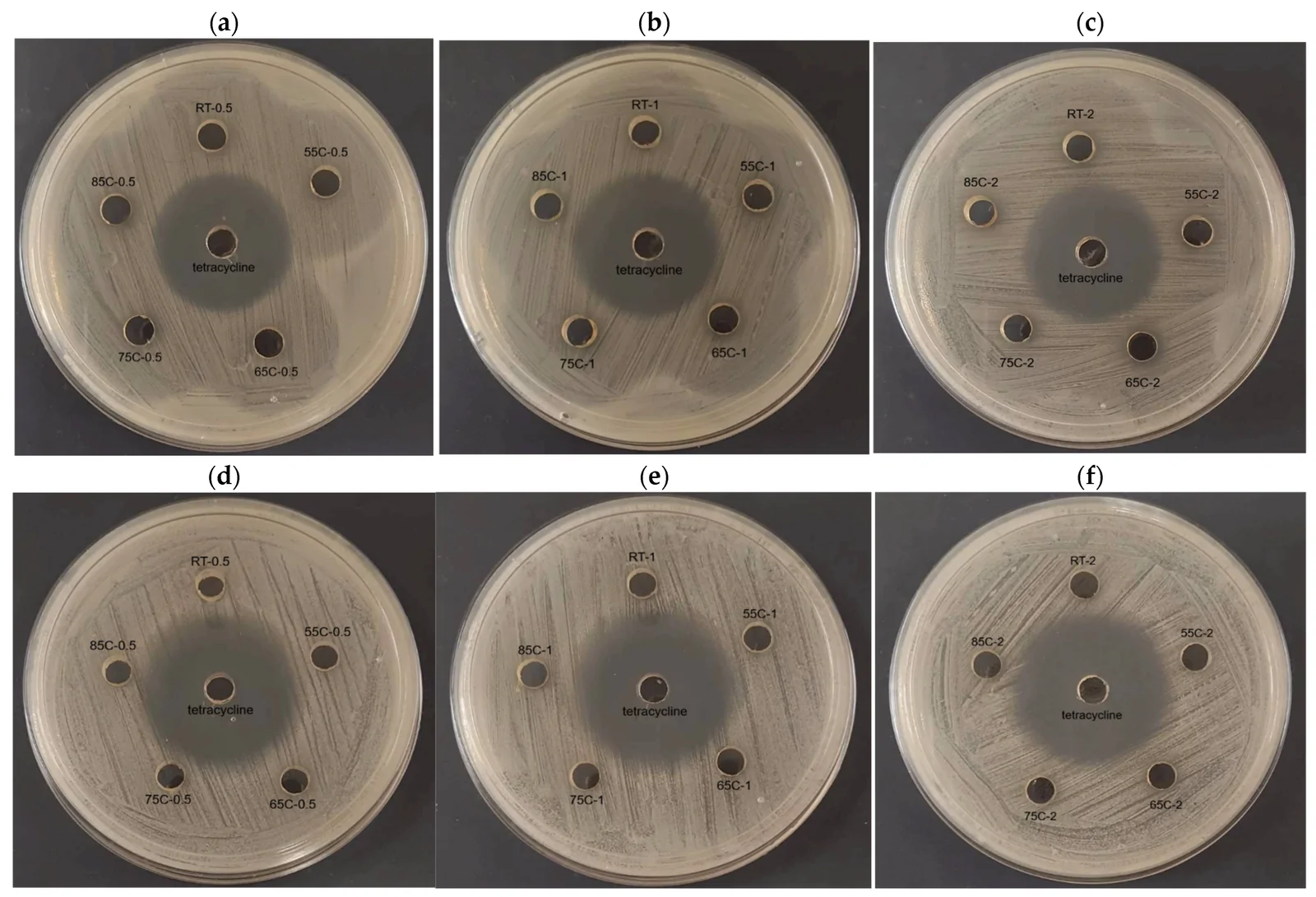
Illustrations of inhibition zones (mm) from antibacterial activity using the agar well diffusion method of *A. dorsata* honey from the fifteen treatment groups against *E. coli* ATCC 25922 (**a**–**c**) and *S. aureus* ATCC 6538 (**d**–**f**). RT-0.5, RT-1, and RT-2 (room temperature for 0.5, 1, and 2 h); 55C-0.5, 55C-1, and 55C-2 (55 °C for 0.5, 1, and 2 h); 65C-0.5, 65C-1, and 65C-2 (65 °C for 0.5, 1, and 2 h); 75C-0.5, 75C-1, and 75C-2 (75 °C for 0.5, 1, and 2 h); 85C-0.5, 85C-1, and 85C-2 (85 °C for 0.5, 1, and 2 h). Tetracycline (1 μg/μL) was used as a positive control.

*Apis dorsata* honey collected from Samut Songkhram province, Thailand, showed antibacterial activity against the Gram-negative bacterium *Escherichia coli* ATCC 25922 but did not show antibacterial activity against the Gram-positive bacterium *Staphylococcus aureus* ATCC 6538 (Figure 3 and Table 2).

The inhibition zone diameter (mm) against *E. coli* ATCC 25922 of *A. dorsata* honey (mean ± SE) heated at all three duration times (0.5, 1, and 2 h) ranged from 7.72 ± 0.19 to 8.06 ± 0.15 mm. Moreover, the inhibition zone diameter (mm) of positive control tetracycline (1 µg/µL) against *E. coli* ATCC 25922 was 27.89 ± 0.2 mm. See Table 2 for statistical comparisons between the groups within a duration time of heat exposure. All honey samples exhibited the same MIC (250 µg/mL) and MBC (500 µg/mL) values against *E. coli* ATCC 25922, indicating that thermal treatment did not alter the inhibitory or bactericidal potency of the honey. In contrast, none of the honey samples demonstrated antibacterial activity against *S. aureus* ATCC 6538. No inhibition zones were detected (0.00 ± 0.00 mm) for any treatment, and both the MIC and MBC values were >500 µg/mL, indicating that the tested concentrations were insufficient to inhibit or kill *S. aureus* ATCC 6538. These results suggest that the antibacterial effect of the honey samples was selective toward the Gram-negative bacterium *E. coli* under the experimental conditions.

The positive control, tetracycline (1 µg/µL), exhibited significantly greater antibacterial activity than all honey samples. It produced inhibition zones of 27.89 ± 0.20 mm against *E. coli* ATCC 25922 and 32.22 ± 0.22 mm against *S. aureus* ATCC 6538, confirming the validity and sensitivity of the assay.

## 4. Discussion

Thermal processing is widely applied in honey handling to improve fluidity, delay crystallization, and facilitate processing; however, it can cause complex effects on honey quality and biological properties. Our study, controlled heating of *A. dorsata* honey (room temperature, 55 °C, 65 °C, 75 °C, and 85 °C for timings of 0.5, 1, and 2 h) produced consistent, temperature–time-dependent changes in color, antioxidant activity, total phenolic contents, total flavonoid contents, and 5-HMF formation. Our findings highlight the dual role of heat in enhancing functional properties while simultaneously promoting chemical degradation.

Honey color (mm Pfund) can be used to indicate the presence of brown pigments by the Maillard reaction [28,29]. The results showed that heating at 85 °C, 75 °C, and 65 °C for all three duration times (0.5, 1, and 2 h) significantly increased the mm Pfund scales (*p* < 0.0001). However, the color of all treatments was identified as light amber (Figure 1) using the criteria following the U.S. Department of Agriculture classifications. The correlation between high brown pigments in honey and high flavonoids and phenolics, contributing to the total antioxidant capacity, was observed in this study, which is consistent with previous reports demonstrating that darker honeys typically contain high levels of phenolic compounds and exhibit high antioxidant properties [17,40]. The honey color intensity comes from nectar sources, geographical origins, and environmental factors influencing chemical composition [41]. Heating or prolonged storage can impact color intensity through the Maillard reaction [42], which is presented in our results (Figure 1 and Table 1).

The effects of thermal treatment on TPC and TFC in our study revealed that heating at 85 °C, 75 °C, and 65 °C for all three duration times (0.5, 1, and 2 h) significantly increased phenolic and flavonoid contents (*p* < 0.0001). The mechanisms by which heat can increase the TPC and TFC are not clear, but one reason that may explain these results is that thermal processing may release bound phenolic compounds from honey matrix components (proteins, polysaccharides) or cell wall fragments present in honey, thereby increasing the extractability and colorimetric detection of phenolics [42,43].

Previous studies showed that heating honey at 75 °C and 95 °C for 0.5 h, 1 h, and 2 h produced higher total phenols and flavonoids than unheated honey [24,44]. Notably, short moderate heating (55 °C for 0.5, 1, and 2 h) often produces little change or non-significant differences in TPC and TFC for many samples, indicating a threshold effect with larger changes occurring at higher temperatures or longer durations [45]. Our results agreed with these previous studies. Interestingly, our results showed TPC of *A. dorsata* honey is 563.22 ± 10.75 mg GAE/100 g. These findings indicate that Thai *A. dorsata* honey has notably higher phenolic contents compared to Indonesian *A. dorsata* honey (248.89 ± 0.57 mg GAE/100 g) [12], Malaysian Gelam honey (78.43 mg GAE/100 g) [9] and comparable levels to Malaysian Tualang honey (25.17 mg GAE/100 g) [46]. Moreover, our results showed the TPC of *A. dorsata* honey is 474.04 ± 12.95 mg QE/100 g. These findings indicate that Thai *A. dorsata* honey has notably higher phenolic contents compared to Indonesian *A. dorsata* honey (88.55 ± 0.93 mg QE/100 g) [12]. Variations in TPC and TFC levels among the samples come from botanical sources and regional differences in bioactive compounds.

The antioxidant activities of *A. dorsata* honey heated at 85 °C, 75 °C, and 65 °C at all three duration times (0.5, 1, and 2 h) significantly increased antioxidant activity (*p* < 0.0001), as indicated by increased %DPPH radical scavenging activity and decreased EC_50_ values [37,47]. Our results revealed that the characteristic antioxidant activities showed a positive correlation with total phenolic and total flavonoid contents, which are high TPC and TFC, and high antioxidant activity [42,48,49]. Additionally, thermal processing promotes the formation of Maillard reaction products, such as melanoidins, which strongly correlated with antioxidant activity [50,51]. The observed increase in antioxidant activity is also consistent with previous studies reporting enhanced antioxidant potential in thermally treated honey due to phenolic and flavonoid enrichment [45]. However, excessive heating, such as 95 °C for 5 min, may lead to degradation of heat-sensitive antioxidants, suggesting that the improvement occurs within an optimal thermal range [52]. Overall, our findings confirm that controlled heating at 85 °C, 75 °C, and 65 °C for 0.5, 1, and 2 h can enhance antioxidant properties.

5-hydroxymethylfurfural content of *A. dorsata* honey heated at 85 °C, 75 °C, and 65 °C at all three duration times (0.5, 1, and 2 h) significantly increased when compared with that of unheated honey (*p* < 0.0001). Thermal processing significantly increased 5-HMF content, confirming progressive honey deterioration [53]. 5-HMF is a Maillard reaction product that can be formed when honey is heated for a long time [26,27,54]. In addition, 5-HMF can also be formed by the dehydration of sugars in an acidic environment, such as honey [28,29].

Our results showed that the highest 5-HMF content of *A. dorsata* honey was found in honey heated at 85 °C (85C-2) with 58.86 ± 1.51 mg/kg; however, it still reached the standard by Codex Alimentarius Commission (the 5-HMF content of honey origin from countries with tropical ambient temperatures, including Thailand, the 5-HMF content shall not be more than 80 mg/kg) [31]. From a regulatory and safety perspective, excessive 5-HMF accumulation is undesirable, as it may compromise nutritional quality and has been associated with potential cytotoxic and mutagenic effects [32,33,34]. Therefore, the increase in 5-HMF underscores the need to carefully control heating conditions during honey processing.

The correlation analysis demonstrated that temperature was the primary factor affecting honey quality during thermal treatment, whereas heating time (0.5–2 h) had only weak effects. Temperature showed very strong positive correlations with color, total phenolic content (TPC), and 5-HMF, and a very strong negative correlation with EC_50_, indicating that higher temperatures enhanced antioxidant activity while promoting browning and 5-HMF formation. Similar findings have been reported in previous studies, where thermal processing accelerated Maillard reactions and sugar degradation, resulting in increased formation of antioxidant compounds, such as melanoidins and other Maillard reaction products [20,24,25,26,27,42]. The strong positive correlations among color, TPC, TFC, and 5-HMF suggest that these parameters are closely associated during heating. Increased color intensity is commonly attributed to the accumulation of flavonoids, phenolic compounds, and Maillard reaction products, both of which contribute to the antioxidant capacity of honey [24,25,26,27,55]. It should be noted that the colorimetric determination of TPC and TFC in honey may be affected by the inherent color of the honey matrix. This consideration is particularly relevant to heat-treated honey because thermal processing can promote Maillard reactions and the formation of colored compounds, which may contribute to the measured absorbance [55,56,57]. Sultana et al. (2024) demonstrated that some TFC values reported for honey may reflect the inherent color of honey rather than exclusively the formation of flavonoid–Al^3+^ coordination complexes [55]. Moreover, the Folin–Ciocalteu method significantly overestimates the TPC since the Folin–Ciocalteu reagent reacts strongly with the melanoidin fraction [56,57]. Therefore, the increases in TPC and TFC observed in the present study should be interpreted cautiously as changes in the apparent colorimetric responses rather than definitive evidence of an increase in the concentrations of phenolic and flavonoid compounds. Furthermore, the strong negative correlations between EC_50_ and TPC, TFC, and 5-HMF indicate that increased antioxidant activity is associated with higher concentrations of phenolics, flavonoids, and heat-induced compounds. Overall, these results suggest that controlled heating enhances the antioxidant potential of honey through the combined effects of naturally occurring phenolic compounds and Maillard reaction products, although excessive heating may increase 5-HMF, an indicator of honey quality deterioration.

The antibacterial activity results further reveal the selective effects of thermal treatment on honey bioactivity. All treated samples exhibited inhibitory activity against the Gram-negative bacterium, *E. coli*, whereas no inhibition was observed against the Gram-positive bacterium, *S. aureus*. The antibacterial properties of honey are attributed to multiple factors, including osmotic pressure, low pH, the presence of phenolic and flavonoid compounds and hydrogen peroxide (H_2_O_2_) production, which can be destroyed easily by heat [58,59,60]. Our results showed that all treatments showed antibacterial activity against *E. coli*, suggesting that these antimicrobial components remain stable after heating.

Phenolic and flavonoid compounds, which increased with temperature in this study, are known to disrupt bacterial membranes and interfere with essential cellular processes, particularly in Gram-negative bacteria [61,62]. Gram-negative bacteria possess a comparatively thin peptidoglycan layer located between the inner cytoplasmic membrane and outer membrane [63]. Although Gram-negative bacteria contain an outer membrane enriched with lipopolysaccharides, the thinner peptidoglycan structure may render them more vulnerable to oxidative damage and membrane disruption caused by honey-derived bioactive compounds such as phenolic and flavonoid compounds [63]. These compounds destabilize bacterial membranes, increase permeability, and interfere with essential enzymatic systems, ultimately inhibiting bacterial growth [61,62]. In contrast, the absence of inhibition against *S. aureus* may be due to Gram-positive bacteria possessing a thick, multilayered peptidoglycan cell wall, typically ranging from 20–80 nm in thickness, which provides substantial mechanical strength and protection against external antimicrobial agents [64,65]. This dense peptidoglycan matrix can act as a physical barrier that limits the penetration of bioactive compounds present in honey, including phenolics, flavonoids, hydrogen peroxide, and other antimicrobial substances [64,65].

Overall, the findings of this study demonstrate a clear trade-off between functional enhancement and quality deterioration in thermally processed *A. dorsata* honey. While heating improves antioxidant-related properties through increased phenolic and flavonoid content and Maillard reaction product formation, it also accelerates 5-HMF accumulation and alters antibacterial activity. These results emphasize the importance of optimizing temperature–time conditions to achieve a balance between enhancing bioactivity and preserving honey quality. Additionally, our study used honey collected from only one geographical region and one harvest season, which may limit the generalizability of the findings. Future studies involving honey from multiple geographical regions and harvest seasons are needed to confirm and extend our results.

## 5. Conclusions

Thermal processing significantly influences the physicochemical properties, bioactive compounds, and antibacterial activity of *A. dorsata* honey. Heating enhanced total phenolic and flavonoid contents, leading to improved antioxidant capacity, and was accompanied by increased color intensity due to the Maillard reaction. However, these beneficial effects were paralleled by a marked increase in 5-HMF, indicating progressive thermal degradation. Our study showed that *A. dorsata* honey heated at 65 °C, 75 °C, and 85 °C for timings of 0.5, 1, and 2 h is the optimal condition, which increases the biological properties without loss in food quality or food safety. Antibacterial activity was retained against Gram-negative bacteria, *E. coli* ATCC 25922, across all treatments, whereas no inhibition was observed against *S. aureus* ATCC 6538, demonstrating selective antimicrobial efficacy. Overall, the results highlight a clear temperature–time-dependent trade-off between functional enhancement and quality deterioration. Optimizing thermal conditions is therefore essential to maximize bioactive properties while minimizing 5-HMF formation and preserving the overall quality of *A. dorsata* honey.

## Figures and Tables

**Table 1:** Biological activities of *A. dorsata* honey from the fifteen treatment groups. RT-0.5, RT-1, and RT-2 (room temperature for 0.5, 1, and 2 h); 55C-0.5, 55C-1, and 55C-2 (55 °C for 0.5, 1, and 2 h); 65C-0.5, 65C-1, and 65C-2 (65 °C for 0.5, 1, and 2 h); 75C-0.5, 75C-1, and 75C-2 (75 °C for 0.5, 1, and 2 h); 85C-0.5, 85C-1, and 85C-2 (85 °C for 0.5, 1, and 2 h). The means ± S.E. with different letters are significantly different from one another. Each group was composed of one cage from each of the three source colonies (three replicate cages per group).

Sample	Color * (mm Pfund)	TPC ** (mg GAE/100 g)	TFC *** (mg QE/100 g)	EC_50_ **** (mg/mL)	5-HFM ***** (mg/kg)
RT-0.5	64.69 ± 1.25 ^g^	554.79 ± 7.16 ^j^	492.53 ± 6.88 ^fg^	81.18 ± 0.57 ^a^	34.53 ± 0.66 ^e^
55C-0.5	64.92 ± 0.73 ^g^	569.92 ± 10.52 ^j^	480.44 ± 6.80 ^g^	78.98 ± 0.92 ^ab^	35.54 ± 0.77 ^e^
65C-0.5	71.53 ± 0.77 ^f^	679.12 ± 11.47 ^h^	525.96 ± 25.36 ^ef^	67.29 ± 2.17 ^c^	45.12 ± 0.84 ^d^
75C-0.5	74.20 ± 1.41 ^ef^	773.18 ± 13.26 ^g^	656.12 ± 14.46 ^d^	63.57 ± 0.72 ^cd^	49.18 ± 1.49 ^c^
85C-0.5	78.73 ± 0.43 ^c^	875.86 ± 2.83 ^e^	615.58 ± 16.89 ^de^	62.67 ± 0.37 ^cd^	51.6 ± 1.76 ^bc^
RT-1	64.25 ± 0.87 ^g^	563.22 ± 10.75 ^j^	491.28 ± 7.08 ^fg^	79.02 ± 2.58 ^ab^	34.71 ± 0.78 ^e^
55C-1	63.95 ± 0.53 ^g^	606.9 ± 10.41 ^i^	504.62 ± 19.39 ^fg^	76.67 ± 1.03 ^b^	35.93 ± 0.93 ^e^
65C-1	72.86 ± 0.84 ^ef^	822.99 ± 9.2 ^f^	615.58 ± 8.97 ^de^	63.72 ± 2.2 ^cd^	45.1 ± 1.06 ^d^
75C-1	76.80 ± 0.35 ^d^	921.26 ± 5.06 ^c^	1217.28 ± 20.26 ^c^	62.67 ± 0.71 ^cd^	50.65 ± 0.36 ^bc^
85C-1	84.75 ± 1.61 ^ab^	1004.22 ± 4.08 ^b^	1407.89 ± 22.81 ^b^	56.08 ± 1.74 ^de^	56.84 ± 1.47 ^ab^
RT-2	64.25 ± 0.86 ^g^	547.51 ± 2.3 ^j^	474.04 ± 12.95 ^g^	81.02 ± 1.83 ^ab^	34.15 ± 0.69 ^e^
55C-2	65.66 ± 0.73 ^g^	623.95 ± 4.67 ^i^	475.46 ± 35.22 ^g^	78.12 ± 2.52 ^ab^	36.99 ± 1.61 ^e^
65C-2	74.42 ± 0.34 ^e^	894.64 ± 6.19 ^d^	649.72 ± 16.22 ^d^	60.57 ± 1.66 ^d^	50.07 ± 1.68 ^c^
75C-2	79.10 ± 0.32 ^bc^	990.61 ± 14.0 ^b^	1163.94 ± 13.29 ^c^	61.04 ± 0.68 ^d^	53.92 ± 1.67 ^bc^
85C-2	85.19 ± 0.67 ^a^	1098.08 ± 9.86 ^a^	1837.48 ± 5.95 ^a^	51.31 ± 0.77 ^e^	58.86 ± 1.51 ^a^

Statistical analysis values: * (Kruskal–Wallis test; *χ*^2^ = 67.29, *df* = 14, *p* < 0.0001); ** (One-way ANOVA test; *F* = 449.8, *df* = 14, *p* < 0.0001); *** (Kruskal–Wallis test; *χ*^2^ = 65.61, *df* = 14, *p* < 0.0001); **** (Kruskal–Wallis test; *χ*^2^ = 64.69, *df* = 14, *p* < 0.0001); ***** (One-way ANOVA test; *F* = 54.89, *df* = 14, *p* < 0.0001).

**Table 2:** Antibacterial activities of *A. dorsata* honey from the fifteen treatment groups. RT-0.5, RT-1, and RT-2 (room temperature for 0.5, 1, and 2 h); 55C-0.5, 55C-1, and 55C-2 (55 °C for 0.5, 1, and 2 h); 65C-0.5, 65C-1, and 65C-2 (65 °C for 0.5, 1, and 2 h); 75C-0.5, 75C-1, and 75C-2 (75 °C for 0.5, 1, and 2 h); 85C-0.5, 85C-1, and 85C-2 (85 °C for 0.5, 1, and 2 h). The means ± S.E. with different letters are significantly different from one another. Each group was composed of one cage from each of the three source colonies (three replicate cages per group).

Sample	Antibacterial Activity Assay					
	*Escherichia coli* ATCC 25922			*Staphylococcus aureus* ATCC 6538		
	Inhibition Zone (mm) *	MIC (µg/mL)	MBC (µg/mL)	Inhibition Zone (mm)	MIC (µg/mL)	MBC (µg/mL)
RT-0.5	7.72 ± 0.19 ^b^	250	500	0.00 ± 0.00	˃500	˃500
55C-0.5	7.94 ± 0.13 ^b^	250	500	0.00 ± 0.00	˃500	˃500
65C-0.5	7.89 ± 0.18 ^b^	250	500	0.00 ± 0.00	˃500	˃500
75C-0.5	7.94 ± 0.06 ^b^	250	500	0.00 ± 0.00	˃500	˃500
85C-0.5	7.89 ± 0.18 ^b^	250	500	0.00 ± 0.00	˃500	˃500
RT-1	7.89 ± 0.18 ^b^	250	500	0.00 ± 0.00	˃500	˃500
55C-1	7.89 ± 0.07 ^b^	250	500	0.00 ± 0.00	˃500	˃500
65C-1	7.78 ± 0.15 ^b^	250	500	0.00 ± 0.00	˃500	˃500
75C-1	7.94 ± 0.06 ^b^	250	500	0.00 ± 0.00	˃500	˃500
85C-1	7.78 ± 0.17 ^b^	250	500	0.00 ± 0.00	˃500	˃500
RT-2	7.78 ± 0.12 ^b^	250	500	0.00 ± 0.00	˃500	˃500
55C-2	7.89 ± 0.14 ^b^	250	500	0.00 ± 0.00	˃500	˃500
65C-2	7.88 ± 0.18 ^b^	250	500	0.00 ± 0.00	˃500	˃500
75C-2	7.92 ± 0.16 ^b^	250	500	0.00 ± 0.00	˃500	˃500
85C-2	8.06 ± 0.15 ^b^	250	500	0.00 ± 0.00	˃500	˃500
tetracycline (1 µg/µL)	27.89 ± 0.2 ^a^	NT	NT	32.22 ± 0.22	NT	NT

Statistical analysis values: * (Kruskal–Wallis test; *χ*^2^ = 28.97, *df* = 15, *p* = 0.0009). Abbreviations: NT = not tested.

## Data Availability

All the data that support the findings of this study are available in the main text.

## Supplementary Materials

The following supporting information can be downloaded at: https://www.mdpi.com/article/10.3390/life16081366/s1.
