# Supplementary material for: The Effect of Temperature Variations on the 5-Hydroxymethylfurfural Content, Antioxidant and Antimicrobial Properties of Honey
Source: Life (Basel). 2026 Aug 19;16(8):1366. doi: 10.3390/life16081366 (PMC13514261; doi:10.3390/life16081366)
Supplement: Supplementary file 1 [file life-16-01366-s001.zip › life-4499535-supplementary.pdf]

Supplementary data

S1. DPPH radical scavenging assay: %DPPH inhibition

The percentage of DPPH inhibition increased over time after heat exposure. The %DPPH inhibition of *A. dorsata* honey heated at 85°C and 75°C was significantly higher than that of room temperature at all three duration times (0.5, 1, and 2 hours) ($\chi^2 = 60.49$, $df = 14$, $p < 0.0001$; Table S1).

The highest %DPPH inhibition of *A. dorsata* honey (mean ± SE) at 0.5 hour duration time of heat exposure was found in honey heat at 85°C (85C-0.5) with 50.08 ± 0.47 % followed by honey from 75C-0.5, 65C-0.5, RT-0.5, and 55C-0.5, respectively. The same general trend continued at 1 and 2 hours of heat exposure. See Table S1 for statistical comparisons between the groups within a duration time of heat exposure.

Table S1. The percentage of DPPH inhibition of *A. dorsata* honey from the fifteen treatment groups. RT-0.5, RT-1, and RT-2 (room temperature for 0.5, 1, and 2 hours); 55C-0.5, 55C-1, and 55C-2 (55°C for 0.5, 1, and 2 hours); 65C-0.5, 65C-1, and 65C-2 (65°C for 0.5, 1, and 2 hours); 75C-0.5, 75C-1, and 75C-2 (75°C for 0.5, 1, and 2 hrs.); 85C-0.5, 85C-1, and 85C-2 (85°C for 0.5, 1, and 2 hours). The means ± S.E. with different letters are significantly different from one another. Each group was composed of one cage from each of the three source colonies (three replicate cages per group).

Sample	%DPPH inhibition
RT-0.5	44.94 ± 0.44 ^{d}
55C-0.5	43.38 ± 0.78 ^{d}
65C-0.5	46.26 ± 0.72 ^{cd}
75C-0.5	49.04 ± 0.46 ^{bc}
85C-0.5	50.08 ± 0.47^{ab}
RT-1	44.92 ± 0.43 ^{d}
55C-1	44.42 ± 0.8 ^{d}
65C-1	48.57 ± 0.82 ^{bc}
75C-1	49.62 ± 0.47 ^{ab}
85C-1	51.21 ± 0.46 ^{a}
RT-2	44.75 ± 0.5 ^{d}
55C-2	44.28 ± 0.76 ^{d}
65C-2	50.47 ± 1.06 ^{ab}
75C-2	50.77 ± 0.55 ^{ab}
85C-2	51.29 ± 0.56 ^{a}

Statistical analysis values: Kruskal–Wallis test; $\chi^2 = 60.49$, $df = 14$, $p < 0.0001$.
